# Global Analysis of Transcriptional Expression in Mice Exposed to Intermediate Frequency Magnetic Fields Utilized for Wireless Power Transfer Systems

**DOI:** 10.3390/ijerph16101851

**Published:** 2019-05-25

**Authors:** Shin Ohtani, Akira Ushiyama, Machiko Maeda, Keiji Wada, Yukihisa Suzuki, Kenji Hattori, Naoki Kunugita, Kazuyuki Ishii

**Affiliations:** 1 Department of Hygienic Chemistry, Meiji Pharmaceutical University, 2-522-1, Noshio, Kiyose, Tokyo 204-8588, Japan; ohtani.s.aa@niph.go.jp (S.O.); ushiyama.niph@gmail.com (M.M.); khattori@my-pharm.ac.jp (K.H.); ishii@my-pharm.ac.jp (K.I.); 2 Department of Environmental Health, National Institute of Public Health, 2-3-6 Minami, Wako, Saitama 351-0197, Japan; kunugita.n.aa@niph.go.jp; 3 Graduate School of Science and Engineering, Tokyo Metropolitan University, Hachioji 192-0397, Japan; kj-wada@tmu.ac.jp (K.W.); y_suzuki@tmu.ac.jp (Y.S.)

**Keywords:** WPT system, IF-MF exposure, mouse, microarray, health effects

## Abstract

Background: Intermediate frequency magnetic fields (IF-MFs) at around 85 kHz are a component of wireless power transfer systems used for charging electrical vehicles. However, limited data exist on the potential health effects of IF-MFs. We performed a comprehensive analysis of transcriptional expression in mice after IF-MF exposure. Materials and Methods: We developed an IF-MF exposure system to generate a high magnetic flux density (25.3 mT). The system can expose the IF-MF for a mouse whole-body without considering thermal effects. After 10 days (1 h/day) of exposure, a comprehensive expression analysis was performed using microarray data from both the brain and liver. Results: No significant differences in transcriptional expression were detected in the 35,240 probe-sets when controlling the false discovery rate (FDR) under a fold change cutoff >1.5. However, several differential expressions were detected without FDR-adjustment, but these were not confirmed by RT-PCR analysis. Conclusions: To our knowledge, this is the first in vivo study to evaluate the biological effects of IF-MF exposure with an intense magnetic flux density 253 times higher than the occupational restriction level defined by the International Commission on Non-Ionizing Radiation Protection guidelines. However, our findings indicate that transcriptional responses in the living body are not affected under these conditions.

## 1. Introduction

Wireless power transfer (WPT) systems are based on magnetic resonance, and electromagnetic induction methods are expected to be commonly used for charging electric vehicles using intermediate frequency magnetic fields (IF-MFs), including 85 kHz bands. However, there is public concern regarding the health effects of IF-MF exposure because evidence of the potential biological effects of IF-MFs is lacking. To achieve early widespread use of WPT systems, obtaining data on the possible biological effects of IF-MF exposure around 85 kHz bands is needed, particularly from in vivo studies.

Microarray analysis is one appropriate method for exploring biological reactions that depend on changes in genome-wide expression associated with health and disease. Microarray analysis has been included in studies of patients with digestive system cancers [1] and toxicity assessments of radiation therapy to reveal abnormal transcriptional responses to DNA damage [2]. In addition, microarray analysis has been applied to screening studies of abnormal transcriptional changes from exposure to radio frequency electromagnetic fields [3,4]. If biological effects of IF-MF exposure exist, comprehensive expression analyses through microarray experiments should provide important data in the primary evaluation of the health effects of IF-MF exposure.

We have assessed the chick embryo-toxicity for IF-MF (20 kHz, 1.1 mT) [5,6], genotoxicity using microorganisms (20 kHz, 1.1 mT) [7], in vitro chemotaxis and phagocytic ability (23 kHz, 2 mT) [8], and comprehensive expression analyses with human glial cells (23 kHz, 6 mT) [9], but no effects were confirmed in any of these studies. However, we have not performed studies on the in vivo biological effects of IF-MF exposure larger than the occupational basic restriction level (100 µT) because of the lack of an appropriate high-power exposure system for animals. Therefore, in this study, we developed an IF-MF exposure system to enable the generation of extremely high magnetic flux density that is 253 times higher than the basic restriction level for occupational exposure as defined in the International Commission on Non-Ionizing Radiation Protection (ICNIRP) guidelines [10]. Using this exposure system, we comprehensively analyzed transcriptional responses derived from IF-MF exposure in the brain and liver of mice to evaluate the in vivo biological effects of around 85 kHz frequency bands exceeding the occupational basic restriction level.

## 2. Materials and Methods

### 2.1. Animals and Ethics Statement

**Figure 1:**
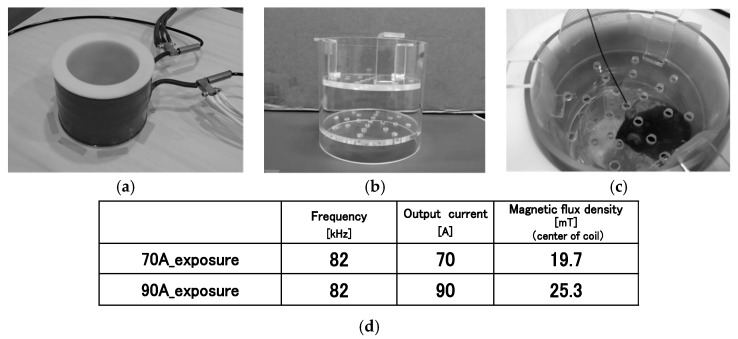
The solenoid coil is shown in (**a**). The acrylic resin holder (**b**) were put into the center space of the coil. A mouse was freely moving in the holder as shown in (**c**). The fiber optic thermometer was set at the top of the acrylic holder containing the mouse. The IF-MF conditions of 70A_exposure and 90A_exposure are shown in (**d**).

Male C57BL/6NCrSlc mice (Japan SLC Inc., Shizuoka, Japan) were used in this study. These mice were housed in an air-conditioned clean room with a 12 h light–dark cycle and given standard chow. For the comprehensive analyses, 15 mice were divided into IF-MF exposed groups consisting of two subgroups, which were 70A_exposure (*n* = 5) and 90A_exposure (*n* = 10) as shown in Figure 1d. When standard deviation, error, and reliability show 0.5%, 1%, and 99%, respectively, the sample size is 5. Fifteen mice were also used for the sham-exposure. Mice were exposed for 1 h/day for 5 days/week. The first exposure was performed on 5-week-old mice. Exposure was sub-chronically continued for 2 weeks (accumulative exposure time, 10 h/mice).

This animal protocol was approved by the Committee of Animal Experiments at the National Institute of Public Health, Japan, under the terms “27-005” and “13 July 2015”.

### 2.2. IF-MF Exposure

**Figure 2:**
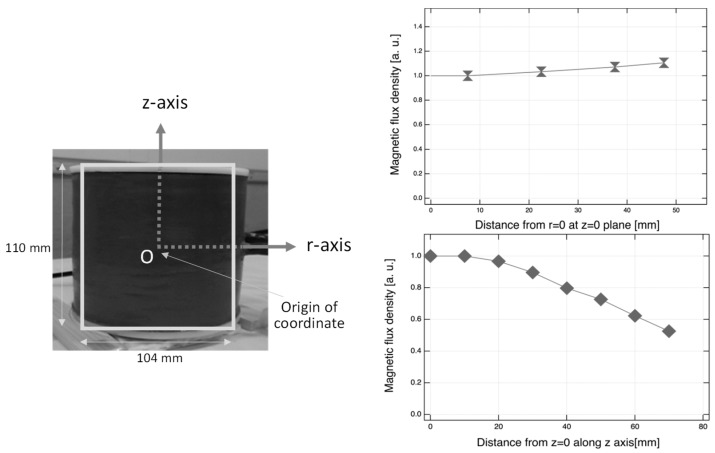
A substantial uniform magnetic flux density was detected in the r-axis direction at the z = 0 plane and in the space of ±20 mm in the *z*-axis direction from the z = 0 plane at the origin of coordinate. The magnetic flux density in each point was determined by the formula of B = (Bx^2^ + By^2^ + Bz^2^)^1/2^.

The IF-MF exposure system used in this study was custom-made and improved from an earlier version [11]. The system was designed to generate high magnetic flux density for the whole body. Briefly, the solenoid coil had the height of 100 mm, the inner diameter of 110 mm, and the structure of 2-layers (14-turns on the inside and 6 winding structure at the top and bottom on the outsides) (Figure 1a). The coil was constructed using a copper pipe connected to an inverter circuit and a cooling water pump. Mice were placed in a custom-made acrylic resin holder (Toyoshima Seisakusho, Tokyo, Japan) (Figure 1b) and put into the center space of the coil without restraint (Figure 1c). As shown in Figure 1d, the magnetic flux density was exposed at 19.7 mT in 70A_exposure and 25.3 mT in 90A_exposure. A magnetic flux density was expressed as a relative value in Figure 2. Magnetic flux density values at measurement points were obtained by 3-axes magnetic probe, which consisted of search coil sensors, and were determined by the formula of B = (Bx^2^ + By^2^ + Bz^2^)^1/2^. The magnetic flux density and a current had a linear relationship. A substantial uniform magnetic flux density was detected in the r-axis direction at z = 0 plane and in the space of ±20 mm in the z-axis direction from the plane of z = 0 (Figure 2). One of the mice was exposed by a uniform magnetic flux density because it was located in this space of ±20 mm using the acrylic holder.

To minimize the thermal effects during IF-MF exposure, excessive heat emitted by the solenoid coil should be suppressed. Therefore, during IF-MF exposure, the temperature around the center of the coil was regulated by water and air cooling to maintain the optimum temperature for a mouse.

### 2.3. Microarray Analysis

After IF-MF exposure, whole brains and livers were extracted from the mice, soaked with RNA later^®^ (Thermo Fisher Scientific, San Jose, CA, USA), and stored at −80 °C. Total RNA was extracted and confirmed for quality (purity, concentration, and degradation). The differential expression of transcripts was assessed in the organs between IF-MF- and sham-exposed mice by using GeneChip^®^ Mouse Gene 2.0 ST Array (Affymetrix, Santa Clara, CA, USA), which covered 35,240 probe sets. Data were analyzed with GeneSpring 14.9 GX software (Agilent Technologies, Palo Alto, CA, USA). The differential expression of transcripts was analyzed using multiple comparison testing with moderated t-testing, with a false discovery rate (FDR) adjusted *p*-value cutoff <0.05 [12] in sham-exposed mice. The fold change cutoff was set to >2.0 or 1.5. Ten mice in the 90A_exposure group were analyzed five by five in 90A_1 and 90A_2 exposure because only five mice could be used per experimental group and also to remove the inter-experimental error.

### 2.4. Analysis with Real Time RT-PCR

Four selected transcripts were analyzed by real time RT-PCR methods following the non-FDR-adjusted approach. The total RNA of the brain and liver was extracted with RNeasy Mini Kits (Qiagen, Hilden, Germany) and reverse-transcribed with High-Capacity cDNA Reverse Transcription Kits (Thermo Fisher Scientific) and the RNA-Quant™ cDNA Synthesis Kit (System Biosciences, Mountain View, CA, USA). The cDNAs were used as templates for the RT-PCR methods. Quantitative PCR was performed with Fast SYBR Green Master Mix (Thermo Fisher Scientific). Primers were designed using Primer3 software (http://frodo.wi.mit.edu/primer3/input.htm) within the amplified fragment length of 150 bp as follows: *Prl*-forward: 5′-ATCAATGACTGCCCCACTTC-3′; *Prl*-reverse: 5′-ACTCGAGGACTGCACCAAAC-3′; *Pomc*-forward: 5′-TTAGCAGATCTGGGGTGGTT-3′; *Pomc*-reverse: 5′-CCTGAGCGACTGTAGCAGA A-3′; *Ftl*-forward: 5′-AGCGTCTCCTCGAGTTTCAG-3′; *Ftl*-reverse: 5′-CTCCTGGGTTTTACCCCATT-3′; and *Mir122*-forward: 5′-TGTGTCCAAACCATC AAACG-3′ (Exigen, Tokyo, Japan). The *Mir122* fragment was amplified with the *Mir122*-forward primer and the universal reverse primer contained in the Kit (RNA-Quant). RT-PCR was performed at 95 °C and 60 °C for 45 cycles in a Stratagene Mx3000P quantitative PCR system (Agilent Technologies, Palo Alto, CA, USA). All samples were assayed in triplicate, and expression data were analyzed with MxPro software (Agilent Technologies). Relative expression levels were determined according to the comparative cycle threshold method using the equation 2^−ΔΔCT^. The average expression of five mice was compared between sham exposure and 70A_ or 90A_exposure, respectively. Statistical analyses were performed with unpaired Student’s *t*-tests (assuming equal variances) or Welch’s *t*-test (assuming unequal variances). Differences were considered significant when *p* < 0.05. 

## 3. Results

### 3.1. Decision of Maximum Exposure Intensity

First, to suppress any thermal effects during IF-MF exposure, the temperature around the top of the acrylic holder containing the mice was measured with the fiber optic thermometer, regulated to 24 °C ± 0.5 °C under both 90A_exposure and sham-exposure groups. Under 100A_exposure, the temperature was uncontrolled by the water cooling, and increased gradually up to 28.5 °C in 1 h. In general, the optimum temperature to rear a mouse is between 21 °C and 25 °C; thus, we decided to perform in vivo exposure experiments at 90A corresponding to 25.3 mT of the magnetic flux density.

### 3.2. Comprehensive Expression Analysis after IF-MF Exposure

Comprehensive expression analysis indicated no differentially expressed transcripts in the brain and liver. These results followed statistical analysis with an FDR-adjusted p-value cutoff of <5% when the fold change cutoff was >1.5 (Table 1). However, this FDR-adjusted statistical analysis may have resulted in the rejection of some true positive responses. Therefore, data were re-analyzed using a moderate t-test approach with non-FDR-adjustment. Under these conditions, several transcripts were identified as altered expressions. In the brain, when the fold change cutoff was set >1.5, the number of altered transcripts of the 70A, 90A_1, and 90A_2 exposure was 6, 9, and 0, respectively (Table 1). Similarly, in the liver, a number of these altered transcripts showed 59, 24, and 6, respectively (Table 1).

### 3.3. Confirmation of the Differential Expression with RT-PCR Analysis

To confirm duplication for the common altered transcripts among the three experiments (70A, 90A_1, and 90A_2 exposure) with the non-FDR-adjusted statistical approach, relative expressions were analyzed with RT-PCR methods. When the selection of transcripts was limited after non-FDR-adjustment by the two satisfied conditions, namely, “the value of the FC cut off >2.0 in at least two experiments out of three experiments” or “the value of FC cut off >1.5 in all three experiments,” we could extract two transcripts each of Mir122 and Ftl1 in the brain and of Prl and Pomc in the liver (Table 2). These relative expressions were analyzed with real time RT-PCR, but no significant changes were detected (Table 3A,B), indicating they were not reproducible.

## 4. Discussion

In the present study, we developed an exposure system for IF-MF that can generate high magnetic flux density in the center of the solenoid coil. Thermal effects derived from the exposure system were suppressed using a water- and air-conditioning system to obtain suitable conditions for the mice. Comprehensive expression analysis revealed no statistically significant differences in the expression of transcripts between IF-MF- and sham-exposed mice following statistical analysis with FDR-adjustment under an FC cutoff >1.5. Indeed, the analyzed transcripts had stable and unchanging expression in the brain and the liver when IF-MF was exposed at 90A_exposrure. Under 90A_ exposure, the whole body value of the induced electric field of the 20.7 g of mouse were 54.1 V/m calculated with the impedance method [13] although it was an unpublished dosimetric calculation. This induced electric field was 2.4 times higher than the basic restriction level (22.9 V/m) for occupational exposure as defined in the ICNIRP guidelines.

We chose to analyze the brain and liver because the central nervous system may be affected by transcriptional and functional changes derived from IF-MF stimulations and the liver plays an essential role in metabolism and detoxication to maintain homeostasis.

Statistical analyses and cutoff values described in McNamee’s report were used [14] to detect subtle transcriptional changes caused by IF-MF exposure. McNamee previously comprehensively analyzed the altered gene expression in mouse brain exposed to a 1.9 GHz radiofrequency. As a result of running multiple comparison testing in the present study, a large number of false-positive events were estimated, at over 1700 out of 35,240 transcripts. On the other hand, the application of FDR-adjusted statistical analysis may have excluded a few true positive responses. To remove statistical errors, we picked up some common altered transcripts following the non-FDR-adjusted approach. We then re-analyzed the selected *Mir122* and *Ftl1* in the brain and *Prl* and *Pomc* in the liver; however, no transcripts were identified as being differentially expressed. These confirmations of reproducibility reinforced that IF-MFs would not affect the differential expression of 35,240 transcripts. Furthermore, dose dependency of the magnetic flux density was not identified between 70A_ and 90A_exposure groups in the number of altered expressed transcripts. If there were a cluster of transcripts showing subtle changes under FC < 1.5, these would be a consideration, but clusters were not detected by clustering analysis of GeneSpring (data not shown). Collectively, these microarray and RT-PCR findings suggest that the threshold level for transcriptional significant changes caused by IF-MF exposure is higher than the occupational restriction level. We assume that the changes are not induced in liver and brain transcripts after two weeks of exposure to IF-MF exposure in the present study.

Some in vivo studies that performed subchronic IF-MF exposure in rats showed no significant changes of teratogenicity, immunotoxicity (21 kHz, 3.8 mT), or developmental toxicity (60 kHz, 0.1 mT) during immature stages [15,16]. Thus, these previous studies and our results indicate that there are little to no biological effects from IF-MF exposure below 100 kHz bands when IF-MF was exposed sub-chronically at the level equal to the basic restriction level or less. Biological assessment from the preceding studies is reinforced by our results as well.

IF-MF exposure could cause biological and functional changes regardless of transcriptional responses. Other indicators should be further studied to understand the whole of the health effects resulting from IF-MF exposure. Our research is ongoing to assess other biological indicators such as stress and behaviors caused by IF-MF exposure. Studies using larger internal induced electrical fields and those evaluating longer term exposure are warranted.

## 5. Conclusions

In conclusion, we developed an IF-MF exposure system for rodents that can generate a high magnetic flux density, which is 253 times higher than the reference level of occupational exposure (100 µT) defined by ICNIRP guidelines. Our results indicate that transcriptional changes in the living body of the investigated animal are not observed in subacute IF-MF exposure at the occupational restriction level or less. The present study is the first in vivo study to evaluate biological effects using a genome wide expression analysis system of IF-MFs utilized in WPT systems.

## Figures and Tables

**Table 1:** Differentially expressed transcripts in the brain and liver between IF-MF- and sham-exposed mice after FDR- and non-FDR-adjusted approaches.

		Brain			Liver		
		70A	90A_1	90A_2	70A	90A_1	90A_2
		N = 5	N = 5	N = 5	N = 5	N = 5	N = 5
FDR-adjusted statistical approach (Benjamini-Hochberg Procedure)	FC > 1.5	0	0	0	0	0	0
Non-FDR-adjusted statistical approach	FC > 2.0	3	4	0	29	2	0
	FC > 1.5	6	9	0	59	24	6

Numbers in this table were the numbers extracted from the 35,240 probe sets under each condition.

**Table 2:** Detected transcripts in the (**A**) brain and (**B**) liver with the following two limitations: the value of FC cutoff of ≥2.0 in at least two out of three experiments (70A, 90A_1, and 90A_2 exposure) or the value of FC cutoff of ≥1.5 in all three experiments after the non-FDR-adjusted approach.

(**A**)						
**Transcripts Cluster Id**	**Brain**					
	**Fold Changes of Expression**			**Gene Symbol**	**Refseq**	**Gene Description**
	**70A**	**90A_1**	**90A_2**			
17286107	**2.34 ↓**	**3.95 ↓**	1.09 ↑	Prl	NM_001163530	Prolactin
17273694	**2.03 ↓**	**2.41 ↓**	1.10 ↓	Pomc	NM_001278581	Pro-opimelanocortin-alpha
(**B**)						
**Transcripts Cluster Id**	**Liver**					
	**Fold Changes of Expression**			**Gene Symbol**	**Refseq**	**Gene Description**
	**70A**	**90A_1**	**90A_2**			
17351321	**2.75 ↓**	**1.58 ↓**	**1.86 ↓**	Mir122	NR_029600	microRNA122
17490830	**2.03 ↓**	**2.18 ↓**	1.10 ↓	Ftl1	NM_010240	Ferritinlightpolypeptide1

If the value of the fold change was >1.5 and 2.0, the notation is shown in boldface. Down or up arrows indicate “down regulation” or “up regulation,” respectively, compared with the sham-exposed group.

**Table 3:** Relative transcript expression levels analyzed with real time RT-PCR in the (**A**) brain and (**B**) liver.

(**A**)									
**Gene Symbol**		**Brain**							
		**70A (N = 5)**				**90A (N = 5)**			
		**Expression (Ave.)**	**Fold Change (Ex/Sh)**	**S.D.**	***t*-Test**	**Expression (Ave.)**	**Fold Change (Ex/Sh)**	**S.D.**	***t*-Test**
*Prl*	Sham	0.80	1.56	±0.70	0.56	15.0	0.84	±19.87	0.85
	Expose	1.26		±1.30		12.64		±12.35	
*Pomc*	Sham	0.36	0.98	±0.34	0.98	2.10	0.77	±1.51	0.67
	Expose	0.36		±0.46		1.62		±1.59	
(**B**)									
**Gene Symbol**		**Liver**							
		**70A (N = 5)**				**90A (N = 5)**			
		**Expression (Ave.)**	**Fold Change (Ex/Sh)**	**S.D.**	***t*-Test**	**Expression (Ave.)**	**Fold Change (Ex/Sh)**	**S.D.**	***t*-Test**
*Mir122*	Sham	0.56	0.61	±0.42	0.38	1.94	0.38	±2.08	0.32
	Expose	0.34		±0.16		0.74		±0.26	
*Ftl1*	Sham	1.00	0.87	±0.39	0.67	1.05	1.00	±0.40	1.00
	Expose	0.87		±0.42		1.05		±0.11	
